# Preliminary Study with the Use of a Titanium Mesh as Space Maker and Implant Primary Stabilization for One-Stage Sinus Lift in Cases with Less Than 1.5 mm Residual Bone

**DOI:** 10.3390/jcm10214853

**Published:** 2021-10-22

**Authors:** Iulian Filipov, Federico Bolognesi, Lucian Chirila, Corina Marilena Cristache, Giuseppe Corinaldesi, Kwang Bum Park

**Affiliations:** 1 Department of Oral and Maxillofacial Surgery, “Queen Maria” Military Emergency Hospital, 9 Pietii Str., 500007 Brasov, Romania; 2 Department of Dental Techniques, Faculty of Midwifery and Medical Assisting (FMAM), “Carol Davila” University of Medicine and Pharmacy, 8, Eroilor Sanitari Blvd, 050474 Bucharest, Romania; 3 Oral and Maxillo-Facial Surgery Unit, IRCCS Policlinico di Sant’Orsola, Via Giuseppe Massarenti, 9, 40138 Bologna, Italy; federico.bolognesi2@gmail.com; 4 Department of Biomedical and Neuromotor Sciences (DIBINEM), University of Bologna, 59 Via S. Vitale, 40100 Bologna, Italy; giuseppe.corinaldesi@unibo.it; 5 Department of Oral and Maxillofacial Surgery, Faculty of Dental Medicine, “Carol Davila” University of Medicine and Pharmacy, 19 Plevnei Ave., 010221 Bucharest, Romania; lucian.chirila@umfcd.ro; 6 Department of Implantology and Periodontology, Daegu Mir Dental Hospital, Jung-gu, Daegu 41934, Korea; periopkb@imegagen.com

**Keywords:** sinus floor augmentation, dental implants, titanium mesh, resonance frequency analysis

## Abstract

(1) Background: In the lateral area of the maxilla, the alveolar bone can lose significant volume due to maxillary sinus pneumatization following teeth extractions. This preliminary study evaluated the effectiveness of a novel technique for one-stage sinus lifting and simultaneous implant placement in cases with less than 1.5 mm residual alveolar bone. The subsequent survival rate at 1-year post-occlusal loading was assessed. (2) Methods: 15 patients were selected, the main inclusion criteria were the partially edentulous area in the posterior maxilla with alveolar bone height of less than 1.5 mm below the sinus. All of the patients underwent one-stage sinus lifting, along with simultaneous implant placement using a “butterfly” anchorage device to optimize the primary stability and xenograft bone as graft material. At 6 to 9 months after surgery, the anchorage device was removed and implants were loaded. Panoramic x-ray images were used to assess the new bone formation, while the biological stability was measured using resonance frequency analysis. (3) Results: 15 implants were inserted. The mean implant stability quotient (ISQ) value was 71.3 (SD = ±2.51) and the mean healing period was 7.3 (SD = ±1.23) months. The mean bone height after the healing period was 14.4 (SD = ±2.05). A statistically significant correlation was found between the healing period and the ISQ value (Spearman rho = 0.684, sig. = 0.005). No statistically significant correlation was found between the ISQ value and the new regenerated bone height (Person r = 0.389, sig. = 0.152). Smoking was identified as a risk factor involved in postoperative complications. (4) Conclusions: The results of the present preliminary study demonstrated that the proposed “butterfly” technique was effective when performing one-stage sinus lifting and simultaneous implant placement in cases with less than 1.5 mm of residual alveolar bone. The survival rate was 100% at 1-year post occlusal loading.

## 1. Introduction

The main goal of implant rehabilitation is the masticatory function. Bone loss is a subsequent consequence of tooth extraction that may undermine the optimal condition of the alveolar bone for implant placement. In the lateral area of the maxilla, the alveolar bone can lose significant volume due to maxillary sinus pneumatization following teeth extractions [1,2]. In many cases, an adjunct augmentation procedure is indicated in order to restore the subantral bone volume. Sinus lifting is a surgical procedure initially described by Boyne and James [3]. The goal of maxillary sinus lifting is to raise the Schneiderian membrane and augment the resulting space with a biomaterial in order to restore the subantral bone height. The prerequisites for this procedure demand a careful pre-operative assessment of the maxillary sinus conditions. It has been shown that conventional radiographic imaging is only 73% reliable [4], while cone beam computed tomography (CBCT) is a more comprehensive and more accurate radiological investigation that may be considered indispensable nowadays, in cases where a sinus lift procedure is planned.

Many anatomical aspects must be considered within the pre-operative treatment planning, such as: membrane thickness, presence of sinus septa, angle between buccal and palatal wall, teeth presence, implants or teeth roots adjacent to the sinus [5]. Any otorhinolaryngological contraindications such as:

− Inflammatory-infective processes, including recurrent or chronic maxillary sinusitis;
− Naso-sinusally located specific systemic granulomatosis diseases;
− Benign and malignant naso-sinus neoplasms involving the maxillary sinus;
− Anatomic-structural impairments of the nasal walls and/or naso-sinus mucosa that may interfere with normal homeostatic naso-sinusal physiology (e.g., post-traumatic or post-surgical scars, sequelae of radiotherapy);

should be detected pre-operatively, and treated conveniently before the sinus lift procedure [4].

Functional endoscopic sinus surgery (FESS) is considered nowadays the current gold standard for many sinusal conditions compliant to surgery.

Schneiderian membrane thickness was suggested to be a significant indicator for the maxillary sinus status by numerous authors [6,7,8]. In order to have concrete indications regarding sinus floor elevation, Tavelli suggested a classification based on the dimensions of Schneiderian membranes, as follows [5]:

− Favorable thickness: 1.5–2.0 mm;
− Normal thickness: 0.8–1.49 mm, 2.01–2.99 mm;
− Unfavorable thickness: <0.79 mm, >3 mm.

If optimal primary stability can be achieved with the simultaneous performance of one-stage sinus lifting and implant placement, this can reduce the number of surgeries, costs, morbidity, and overall treatment duration.

Traditionally, 4–5 mm is considered the minimum necessary subantral height for a one-stage approach [9,10,11]. However, numerous authors have still reported favorable results with less than 4 mm of alveolar bone height [12,13].

The major concerns of the patient for restoring function and aesthetics in the posterior maxillary arch are morbidity and treatment duration.

Several attempts for providing a predictable option for the sinus graft with simultaneous implant placement in severe pneumatization were made, but none proved to be efficient [14]. Therefore, the aim of this prospective study is to apply a novel surgical technique to one-stage sinus lifting and simultaneous implant placement in cases with less than 1.5 mm alveolar bone height, as well as to evaluate the success rate at 1-year after occlusal loading.

## 2. Materials and Methods

**Figure 1:**
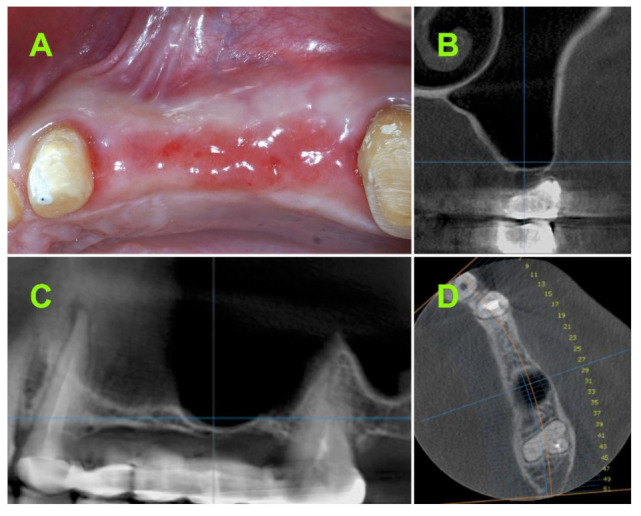
(**A**) Clinical aspect in the lateral area of upper maxilla; Pre-operative CBCT: (**B**) Coronal view; (**C**) sagittal view; (**D**) axial view.

The present study was conducted in accordance with the ethical principles including the World Medical Association Declaration of Helsinki, the Belmont report, the Council for International Organizations of Medical Sciences (CIOMS) guidelines, and the International Conference on Harmonization in Good Clinical Practice (ICH-GCP). The study was conducted in Brasov, Romania and approved by the Bioethical Committee of “Queen Maria” Military Hospital, Brasov, Romania, no. 1926/2020. Moreover, written consent of each subject was obtained. A total of 15 consecutive patients (7 female/8 male) with chief complaint masticatory dysfunction due to one or several missing teeth (Figure 1A) in the lateral area of the maxilla, were selected for this study. The patients were all examined to determine their eligibility for the study.

### 2.1. Inclusion Criteria

#### 2.1.1. Subject Inclusion Criteria

− Age greater than 18;
− Single or multiple edentulous spaces in maxillary lateral area;
− No contraindications for oral surgery related to systemic health;
− Both patients are smokers and non-smokers;

Acceptance of the proposed treatment and follow-up protocol, as well as willingness to sign the consent form.

#### 2.1.2. Study Site Inclusion Criteria

− Residual subantral native bone height of 1.5 mm or less at implant site (based on CBCT measurements);

No pathological condition related to the maxillary sinus.

### 2.2. Study Design

This prospective pilot study was designed to assess the biological stability of implants placed simultaneously with lateral sinus lifting, where the implant primary stability was supported using an adjunctive fixation device.

#### 2.2.1. Pre-Operative CBCT Measurements

Pre-operative CBCT measurements were used as the baseline in all of the cases (Figure 1B–D). All of the CBCT measurements were performed using the measurement tool from the Planmeca Romexis^®^ software, version 3.2.7 (Helsinki, Finland). The pre-operative bone height measurements were performed from the ridge crest to the sinus floor in cross-sectional images of the implant placement site. All of the measurements were assigned into one of the following groups: Less than 0.5 mm residual bone height, between 0.5- and 1-mm residual bone height, and between 1- and 1.5-mm residual bone height.

#### 2.2.2. Surgical Techniques

**Figure 2:**
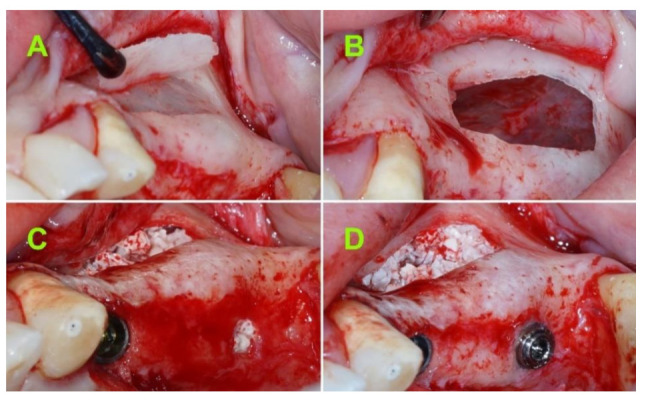
(**A**) Bone lid removal and preservation; (**B**) Schneiderian membrane elevation; (**C**) xenograft material used to fill the submucosal space; and (**D**) implant and flat abutment in transitional position, 2–3 mm above the bone crest.

After local anesthesia, a horizontal incision was made along the middle of the alveolar crest between the neighboring teeth. Then, two vertical-releasing incisions were extended into the alveolar mucosa. A trapezoidal full-thickness flap was elevated and the anterior wall of the maxillary sinus was exposed. All of the soft tissue debris on the surface of the bone were thoroughly removed using a sharp periosteal elevator. The sinus lifting procedure was performed according to the methods previously described by Boyne and James [3]. The bone lid was removed and preserved (Figure 2A), and the maxillary sinus membrane was elevated as necessary (Figure 2B). A lance drill was used to mark the implant site, followed by a 3.3 mm drill (AnyRidge, MegaGen Implant Co., Daegu, Korea) in order to create the osteotomy for implant placement. The implant insertion protocol, bone grafting procedure, and titanium mesh adjustment were described in a previously published paper [15].

After elevating the maxillary sinus mucosa according to the pre-established plan, a resorbable membrane (Collprotect Membrane^®^, Botiss biomaterials GmbH, Berlin, Germany) was placed under the Schneiderian membrane. Then, a mixture of deproteinized bovine bone mineral particles (Cerabone^®^, Botiss GmbH, Berlin, Germany) with a size between 1 and 2 mm mixed with 2 mL of 5 mg/mL metronidazole B solution was used as the graft material (Figure 2C).

A 4.0 mm diameter tapered implant (AnyRidge, MegaGen Implant Co., Daegu, Korea) with either 10 mm or 11.5 mm in length, was inserted in each site using a surgical motor (at 35 RPM) until approximately 0.5 mm below the bone crest. Holding the implant with tweezers can facilitate the removal of the implant connector without the mobilization of the implant.

A 1.5 to 4.5 mm flat abutment (I-Gen Kit, MegaGen, Daegu, Korea) was connected to the implant using digital clockwise rotations (Figure 2D).

A titanium “butterfly” mesh (I-Gen Kit, MegaGen, Daegu, Korea) was then customized on the spot using surgical scissors in order to be adapted to the implant site.

**Figure 3:**
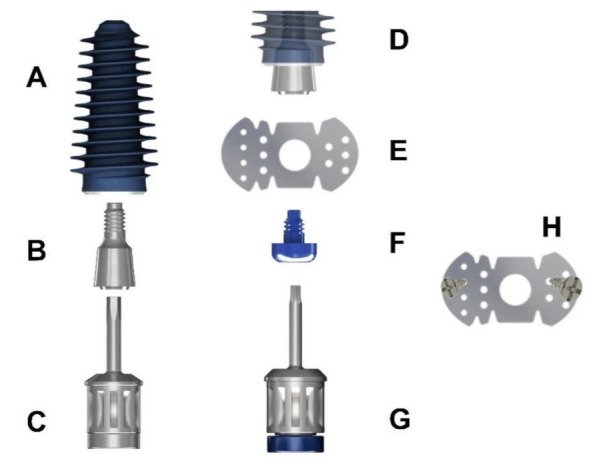
The “butterfly” anchoring device: (**A**) Implant fixture; (**B**) flat abutment screw; (**C**) handheld screwdriver for flat abutment; (**D**) flat abutment in final position; (**E**) titanium mesh; (**F**) cover screw; (**G**) handheld screwdriver for cover screw; (**H**) self-drilling 3 mm screws for titanium mesh fixation.

The “butterfly” anchoring device (Figure 3A–H) is a mixture of one flat abutment (Figure 3B), one titanium mesh (Figure 3E), one cover screw (Figure 3F), and two self-drilling cortical screws (Figure 3H). Two handheld screw drivers are necessary to fix the screws (Figure 3C,G) into the implant (Figure 3A) or flat abutment (Figure 3D).

Attention must be paid to leave at least 1.5 mm from the margins of the mesh to the neighboring teeth in order to avoid postoperative exposure.

**Figure 4:**
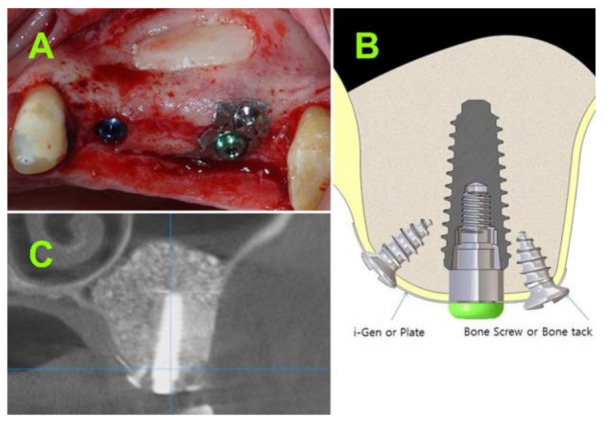
(**A**) Clinical aspect of implant secured with the “butterfly” device; (**B**) schematic drawing of “butterfly” device; and (**C**) CBCT at 7 months postoperative.

A tweezer was used to position the mesh above the flat abutment and keep it in place, while a hand driver was used to connect the cover screw through the mesh into the flat abutment screw. Digital clockwise rotations were executed until the mesh wings reached the surface of the alveolar bone. To secure the “butterfly”, two self-drilling (3 mm) screws were placed through the wings of the mesh into the cortical bone, one from the palatal aspect and one from the labial aspect (Figure 4A,B), thereby achieving optimal stability for the totally “immersed” implant.

Releasing incisions were performed to the periosteum at the base of the muco-periosteal flap to facilitate primary closure. Double layer suturing (horizontal mattress and simple suturing) was performed using 5/0 resorbable sutures (Vicryl, Ethicon, New Brunswick, NJ, USA).

#### 2.2.3. Postsurgical Infection Control

All of the patients were prescribed antibiotics (amoxicillin and clavulanic acid 1 g/every 12 h), in addition to an anti-inflammatory (ibuprofen 600 mg/every 8 h) for 5 days.

Moreover, the patients were instructed to avoid brushing the neighboring teeth in the treated area. A chlorhexidine solution (0.12%) was prescribed for daily usage (twice a day for 1 min). The sutures were removed after 14 days. All of the patients were recalled for a clinical check-up at 1, 3, and 6 months after the sinus lifting.

#### 2.2.4. Healing Abutment Phase

At 6 to 9 months after the surgical procedure, a CBCT (Figure 4C) was performed to assess the bone gain and occurrence of sinus complications.

**Figure 5:**
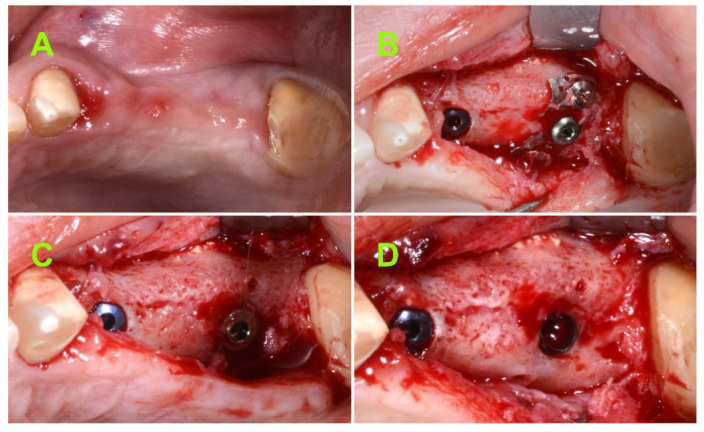
(**A**) Seven months postoperative clinical aspect; (**B**) intra-operative aspect after uncovering the regenerated site; (**C**) intra-operative aspect after removing the membrane and screws; and (**D**) intra-operative aspect after removing the flat abutment screw.

An incision was made along the middle of the crest and two limited full-thickness flaps were raised to expose the mesh (Figure 5A,B). Step-by-step, the self-drilling screws, cover screw, mesh, and flat abutment screw were all removed and the implant was exposed (Figure 5C,D). A SmartPeg was connected to the implant and the biological stability was measured using the Osstell ISQ from two different directions (at 90° angle relative to the implant axis).

Three weeks after healing of the abutment insertion, the prosthetic procedure was initiated. Prefabricated titanium abutments and cemented crowns or three units’ bridges were used to restore the inserted implants. One week after the final crown/bridge insertion, a panoramic x-ray was taken.

**Figure 6:**
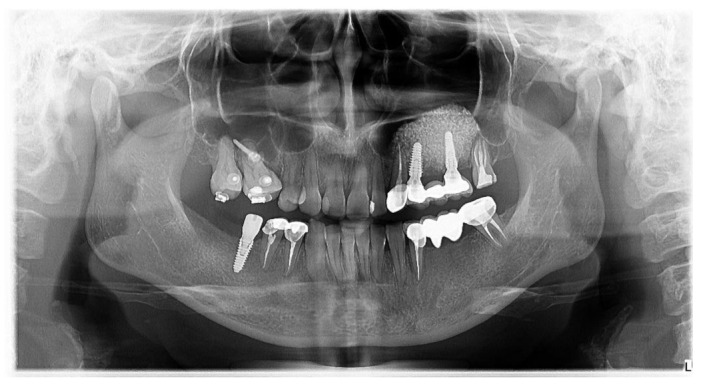
Postoperative panoramic x-ray at 1-year after loading.

Moreover, a second panoramic x-ray was taken for all of the cases at 1-year after loading (Figure 6).

#### 2.2.5. Bone Height Evaluation of Newly Regenerated Bone

At 6 to 9 months after the sinus lift procedure, at the time of healing of the abutment fixation, the measurement tool from the Planmeca Romexis^®^ software, was used to measure the bone height of the regenerated bone from the ridge crest to the newly established sinus floor, in cross-sectional CBCT images, at each implant placed site. For each site, the subantral bone height was considered the mean value of two measurements from the ridge crest to the newly established sinus floor.

#### 2.2.6. Evaluation of 1-Year Bone Remodelling

The Image J open software (https://imagej.nih.gov/; accessed on 13 September 2021), calibrated for each measurement based on the known implant length [16], was used to measure the peri-implant marginal bone level at mesial and distal sites, on the panoramic radiographs at prosthetic insertion and at 1-year post loading. For calibration with the known implant length, the reference points were implant apex and implant collar. In addition, for mesial and distal bone level measurements, the reference points were the correspondent horizontal point of the implant apex and the most coronal bone to the implant/abutment contact. The mean bone level at implant loading for each site was considered baseline. Bone loss at the 1- year follow-up was measured by subtracting the measured mean bone level from the baseline for each site. The mean value for mesial and distal sites was recorded.

#### 2.2.7. Implant Stability Evaluation

Since the time of implant placement or “initial” primary stability was considered 0, no objective measurement of primary stability was performed. At 6 to 9 months after implant placement, at healing of the abutment insertion, the biological stability was assessed using resonance frequency analysis with the aid of an Osstell ISQ device (Integration Diagnostics, Göteborg, Sweden), in an objective and qualitative manner. Two different measurements were conducted using the handheld Osstell ISQ probe. The corresponding SmartPeg for AnyRidge implant was manually screwed onto the implant and the probe was placed approximately 3 mm from the SmartPeg at a 90° angle relative to the implant axis. Two measurements, 90° perpendicular to each other, were performed (mesio-distal and labio-palatal). The final value was calculated as the average of the two measurements.

#### 2.2.8. Complications

All of the complications that occurred during the surgery (Schneiderian membrane perforation) and postoperatively (cover screw or titanium mesh exposure) were reviewed.

#### 2.2.9. Data Analysis

The IBM SPSS statistical software (version 20.0, SPSS, IBM Corp., Armonk, NY, USA) was used for the statistical analysis. A descriptive analysis was performed for assessing the biological stability of the implants and status of the newly formed bone and mean. In addition, the SD was calculated. The Pearson correlation coefficient was used to assess the correlation between the mean ISQ and mean bone height measurements. The normality condition was verified using the Shapiro-Wilk test. Due to the lack of normal distribution, the Spearman correlation coefficient was used to assess the correlation between months of healing with the other variables. Significance was set at a *p*-value < 0.05.

## 3. Results

A total of 15 patients were enrolled in the study, seven female and eight males. The mean age of study participants was 46 years, ranging from 28 to 71 years.

Each patient underwent a sinus lift procedure with the simultaneous placement of an implant in the alveolar bone that was less than 1.5 mm in height.

Overall, 33 implants were placed and stabilized using a “butterfly” titanium mesh. However, as the protocol of the present study was one-stage sinus lifting and simultaneous implant placement in cases with less than 1.5 mm alveolar bone height, only 15 implants fulfilling this criterion were included in the statistical analysis.

In seven cases, 46.6% of implants were placed in less than 0.5 mm of native bone; in five cases, 33.3% of implants were placed in 0.5–1 mm of native bone; and in three cases, 20% of implants were placed in 1–1.5 mm of native bone.

Two (13.3%) implants were placed at the site of the second premolar: Nine (60%) implants at the site of the first molar, and four (26.6%) implants at the site of the second molar.

Four implants were restored with single metal ceramic crowns on prefabricated abutments, three on the first molar site and one on the second molar site. The other 11 implants were restored with metal ceramic three units’ bridges. In most of the cases, the use of the “butterfly” technique was required for both of the placed mesial and distal implants. However, only the implants inserted in native bone height of less than 1.5 mm were included in the statistical analysis.

Out of the 15 patients included in the present study, two were smokers (more than 15 cigarettes a day).

### 3.1. Assessment of Bone Height at Implant Site

The pre-operative bone height and the 6- to 9-month postoperative bone height for the 15 assessed implants are displayed in Table 1.

### 3.2. Evaluation of 1-Year Bone Remodelling

The evaluation of marginal bone loss is displayed in Table 2 and was calculated as the difference between the bone level at 1-week post loading and bone level at 1-year follow-up.

### 3.3. Implant Stability Evaluation

The mean ISQ values before loading are displayed in Table 3. For each measurement, the lowest value of three successive measurements in mesio-distal and labio-palatal directions was considered.

The mean ISQ value was 71.3 (SD ± 2.51) and the mean healing period was 7.3 (SD = ±1.23) months. The mean bone height after the healing period was 14.4 mm (SD = ±2.05). The mean marginal bone loss at 1-year after loading was 0.94 mm (SD = ±0.48).

A statistically significant correlation was found between the healing period and the ISQ value (Spearman rho = 0.684, sig. = 0.005).

Yet, no statistically significant correlation was found between the ISQ value and the new regenerated bone height (Pearson r = 0.389, sig. = 0.152).

### 3.4. Complications

In three (20%) cases, the maxillary sinus membrane was perforated, resulting in a perforation of less than 5 mm. In each case, a resorbable membrane was used to restore the integrity. Notwithstanding, the intra-operative perforation of the Schneiderian membrane showed no influence on the survival or success rate at 1-year after occlusal loading.

There was one case where the cover screw became exposed after 7 weeks, and another case where the cover screw and part of the titanium mesh became exposed after 13 weeks. For the case where only the cover screw became exposed, the maximum longitudinally assessed bone loss was 1.5 mm, while for the case where both the cover screw and the mesh became exposed, the bone loss was 2 mm.

## 4. Discussion

To the author’s knowledge, this is the first study with one-stage implant insertion and sinus grafting in cases with a residual alveolar bone height between 0.5 and 1.5 mm.

Several treatment options have been utilized to overcome the issue of inadequate bone volume in the lateral area of the maxilla [17]. The subantral residual alveolar bone plays a significant role with respect to the most convenient option of surgical procedure and the timing of implant placement. Short implants (defined as implants with an intrabony length of 8 mm or less) [18,19] have been suggested as a favorable option in order to minimize the morbidity and the risk of a more invasive procedure, such as the sinus lift. However, a minimum of 6 mm of residual alveolar bone is necessary for short implant placement. In addition, the subsequent complication rate is influenced by the type of the delivered prosthesis, either splinted or non-splinted.

It was suggested that splinting was a positive factor in the success of very short implants [20]. The residual alveolar bone height and the type of delivered prosthesis constrict the indication for short implant placement only for a limited population.

In order to restore the bony foundation for convenient implant placement, sinus lift was described as a predictable procedure [3,21,22,23,24,25,26].

To date, two main evidence-based techniques of sinus floor elevation for dental implant placement are in use. Tatum described sinus augmentation and implant placement as a one-stage and a two-stage technique [27]. The technique known as a lateral window sinus lift is widely used today and the decision to apply the one or the two-stage technique is based on the amount of subantral residual bone. According to numerous authors, simultaneous and delayed procedures display similar survival rates [13,21,23,24,26].

One-stage sinus lifting was initially indicated in cases with residual alveolar bone measuring at least 4–5 mm [9,28]. Summers considered it necessary to have at least 6 mm of residual bone to ensure the primary stability of the implant. For cases of less than 6 mm of residual bone height, Summers proposed a two-stage approach [22]. Cosci outlined a modified one-stage crestal technique with as little as 3 mm of residual bone and qualified the procedure as highly predictable and reliable [29].

Traditionally, 4 mm was considered a minimal value that would facilitate the achievement of optimal primary stability and thus, qualify a patient for a one-stage or two-stage procedure. However, this is an arbitrary established value that apparently would guarantee a convenient bone volume in order to achieve implant primary stability. Subsequent publications have shown similar final outcomes in cases with 1 to 4 mm of residual alveolar bone height where one-stage sinus lifting was performed [10,12,13,30]. Cha showed that cumulative survival and success rates were 98.91% when the residual alveolar bone height was 4 mm or less and 96.54% alveolar bone height was 5 mm or more [31]. According to a multicenter randomized controlled trial, it was suggested that no statistically significant differences were observed between the implants placed using the one- or two-stage sinus lift procedure [32]. The inclusion criteria for the test group were having 1 to 3 mm of residual bone height and at least 5 mm of bone width below the maxillary sinus, as measured on CT scans.

Chin proposed a technique for “suspending implants in a semirigid fashion” in the center of the reconstruction environment or “bone-forming chamber”, using a 0.3-mm-thick titanium bone plate secured with two micro screws placed buccally and attached to the implant by the cover screw [33]. The technique was applied in different clinical cases with no post loading follow-up. However, the proposed fixations were placed exclusively buccally with a risk of implant bending during healing, if the native bone is missing. Moreover, in some cases, the buccal bone is thin and prone to resorption [34] with consequently threads exposure or loss of implant stability and failure of osseointegration. Furthermore, the bone plate proposed by Chin for implants immobilization is directly attached to the implant with the cover screw [33]. The direct attachment of the fixation device to the implant will lead to excessive pressure on the buccal bone and will restrict the implant insertion depth to the existing level of the crestal bone.

Our proposed “butterfly” device has a double fixation (buccal and palatal) to avoid the excessive pressure on the buccal bone, as well as a 1.5 to 4.5 mm flat abutment allowing for an adequate depth of implant insertion.

In the present study, one inclusion criterion was residual subantral native bone height of 1.5 mm or less at implant site (based on CBCT measurements). Therefore, the recommended treatment protocol according to the last 40 years of practical guidelines, would have been to perform a two-stage approach sinus lift. However, due to the novelty of the approach, the one-stage sinus lift was performed with a survival rate of 100%. In cases with a residual alveolar bone height of less than 1.5 mm, primary implant stability would be highly unpredictable, if not impossible to achieve, without additive procedures (e.g., bone block) [35] or stabilization devices. Volmer [36] and Grandi [37] previously proposed the use of mini-plates for osteosynthesis in order to facilitate simultaneous implant placement. However, their case selection process was not exclusively limited to cases with less than 1.5 mm of residual bone. Moreover, the practicability of these mini-plates is questionable, since, in many clinical scenarios, the alveolar bone with more than 1.5 mm of height can facilitate a simultaneous implant placement without any additional device that might complicate the surgery [12,13]. Furthermore, bending of the osteosynthesis plates is more time consuming, difficult to adapt to the architecture of the bone, and invariably limits second molar implant placement. In contrast, the proposed “butterfly” anchorage relies mainly on the palatal and labial bone. Considering that the residual alveolar bone height was less than 1.5 mm in our clinical cases, placing the implants in a subcrestal manner without any adjunctive device for implant stabilization would not have been possible. Even though the implant may have absolutely no contact with the residual subantral bone after reaching its final position, an optimal primary stability can still be achieved as a result of implant anchoring in the palatal and labial bone.

As the mesh is localized above a flat abutment screw and not on the implant shoulder itself, the implant placement can be performed at different depths below the bone crest, according to the length of the selected flat abutment (from 1.5 to 4.5 mm).

Except for two cases, when the patients were heavy smokers, all of the inserted implants showed an acceptable mean marginal bone loss at 1-year follow-up, according to the success criteria of Albrektsson et al. (>1.5 mm marginal bone loss during the first year of function) [38]. However, the bone level at implant loading was considered baseline. A bone gain was obtained during osseointegration with the use of the “butterfly” titanium mesh. In addition, all of the implants were inserted 0.5 mm below the crestal bone, in an ideal position for prosthetic reconstruction.

Consequently, since two (13.3%) implants did not meet the implant success criteria according to [38], the success rate was 86.6% and the survival rate was 100% at 1-year post-occlusal loading.

The bone graft material used in our study was a xenograft, deproteinized bovine bone. However, the bone formation after the sinus lift procedure is not influenced by the type of bone graft material. In many cases, a space holder, for keeping the Schneiderian membrane elevated and protecting the blood cloth is sufficient for enabling bone regeneration [39,40].

From a surgical point of view, the proposed newly structured titanium anchoring device of the dental implant provided optimal primary stability with a minimal residual alveolar height. In addition, the survival rate was similar to most of the other studies where a two-stage approach was followed.

Despite the limited number of cases treated, the results of the present preliminary study indicate that the proposed technique was highly effective in the treatment of severely atrophic alveolar bone in the maxillary lateral area. Moreover, accidental perforation of the Schneiderian membrane did not restrict the possibility of one-stage sinus lifting, as long as the membrane perforation was restored with a resorbable membrane.

## 5. Conclusions

The following conclusions can be drawn from the present preliminary study:

The “butterfly” technique was shown to be effective for performing one-stage sinus lifting with simultaneous implant placement in cases with less than 1.5 mm of residual alveolar bone height.

The proposed technique is a reliable transitional option to sustain implant stability until mineralization of the graft material creates implant osseointegration.

At 6 to 9 months, all of the placed implants showed optimal biological stability.

At 1-year after occlusal loading, the implant survival rate was 100%.

Additional controlled multicenter clinical trials are needed to confirm the promising results of the present pilot study.

## Figures and Tables

**Table 1:** Measurement of the crestal bone pre-operatively and at 6 to 9 months postoperatively.

	Preoperative Measurements (on CBCT)	Postoperative 1st Measurement	Postoperative 2nd Measurement	Postoperative Mean Values (±SD)
1	<0.5	14	15	14.5 (±0.71)
2	0.5–1	16	16.5	16.2 (±0.35)
3	0.5–1	16	15	15.5 (±0.71)
4	<0.5	13	11	12 (±1.41)
5	1–1.5	17	17	17 (±0)
6	<0.5	12	13	12.5 (±0.71)
7	0.5–1	17	16	16.5 (±0.71)
8	0.5–1	13	14	13.5 (±0.71)
9	<0.5	18	18	18 (±0)
10	<0.5	13	12	12.5 (±0.71)
11	1–1.5	15	14	14.5 (±0.71)
12	<0.5	13	14	13.5 (±0.71)
13	<0.5	11	12	11.5 (±0.71)
14	0.5–1	13	13	13 (±0)
15	1–1.5	16	17	16.5 (±0.71)

Light brown rows—preoperative crestal bone <0.5 mm; grey rows—preoperative crestal bone 0.5–1 mm; white rows—preoperative crestal bone 1–1.5 mm.

**Table 2:** Mean marginal bone loss at 1-year follow-up.

	Mean Bone Level at Loading (±SD)	Mean Bone Level at 1-Year Follow-Up (±SD)	Mean 1-Year Marginal Bone Loss
1	13.48 (±0.36)	13.16 (±0.18)	0.32
2	13.71 (±0.45)	12.75 (±0.32)	0.97
3	14.41 (±0.05)	13.34 (±0.14)	1.07
4	10.95 (±0.62)	10.84 (±0.06)	0.11
5	14.60 (±0.38)	13.39 (±0.40)	1.21
6	12.18 (±0.30)	11.25 (±0.36)	0.93
7	13.73 (±0.55)	12.45 (±0.33)	1.28
8	12.90 (±0.08)	10.90 (±0.09)	2.00
9	14.56 (±0.47)	13.33 (±0.17)	1.23
10	12.14 (±0.71)	11.36 (±0.02)	0.78
11	14.38 (±0.06)	12.88 (±0.13)	1.50
12	12.64 (±0.12)	11.71 (±0.20)	0.93
13	10.80 (±0.60)	10.23 (±0.06)	0.57
14	12.44 (±0.06)	11.64 (±0.33)	0.80
15	15.35 (±0.31)	14.97 (±0.14)	0.38

Light brown rows—preoperative crestal bone <0.5 mm; grey rows—preoperative crestal bone 0.5–1 mm; white rows—preoperative crestal bone 1–1.5 mm.

**Table 3:** Measurement of the ISQ values 6 to 9 months postoperatively, before implants loading.

	Mesio-Distal ISQ Measurement	Labio-Palatal ISQ Measurement	Mean ISQ Values (±SD)
1	73	71	72 (±1.41)
2	76	74	75 (±1.41)
3	68	71	69.5 (±2.12)
4	70	73	71.5 (±2.12)
5	73	72	72.5 (±0.71)
6	69	68	68.5 (±0.71)
7	71	71	71 (±0)
8	72	69	70.5 (±2.12)
9	74	76	75 (±1.41)
10	76	75	75.5 (±0.71)
11	70	68	69 (±1.41)
12	71	72	71.5 (±0.71)
13	69	72	70.5 (±2.12)
14	68	65	66.5 (±2.12)
15	70	74	72 (±2.83)

ISQ: Implant stability quotient; Light brown rows—preoperative crestal bone <0.5 mm; grey rows—preoperative crestal bone 0.5–1 mm; white rows—preoperative crestal bone 1–1.5 mm.

## Data Availability

Data are available from the corresponding authors upon request.
